# Supplementary material for: Association between oral microbiome and seven types of cancers in East Asian population: a two-sample Mendelian randomization analysis
Source: Front Mol Biosci. 2023 Nov 21;10:1327893. doi: 10.3389/fmolb.2023.1327893 (PMC10702768; doi:10.3389/fmolb.2023.1327893)
Supplement: Supplementary file 1 [file DataSheet1.ZIP › Supplementary Materials/MR plots for tongue/tongue═╝/Ovarian cancer/pheno.3117_to_ovarian cancer_forest.pdf]

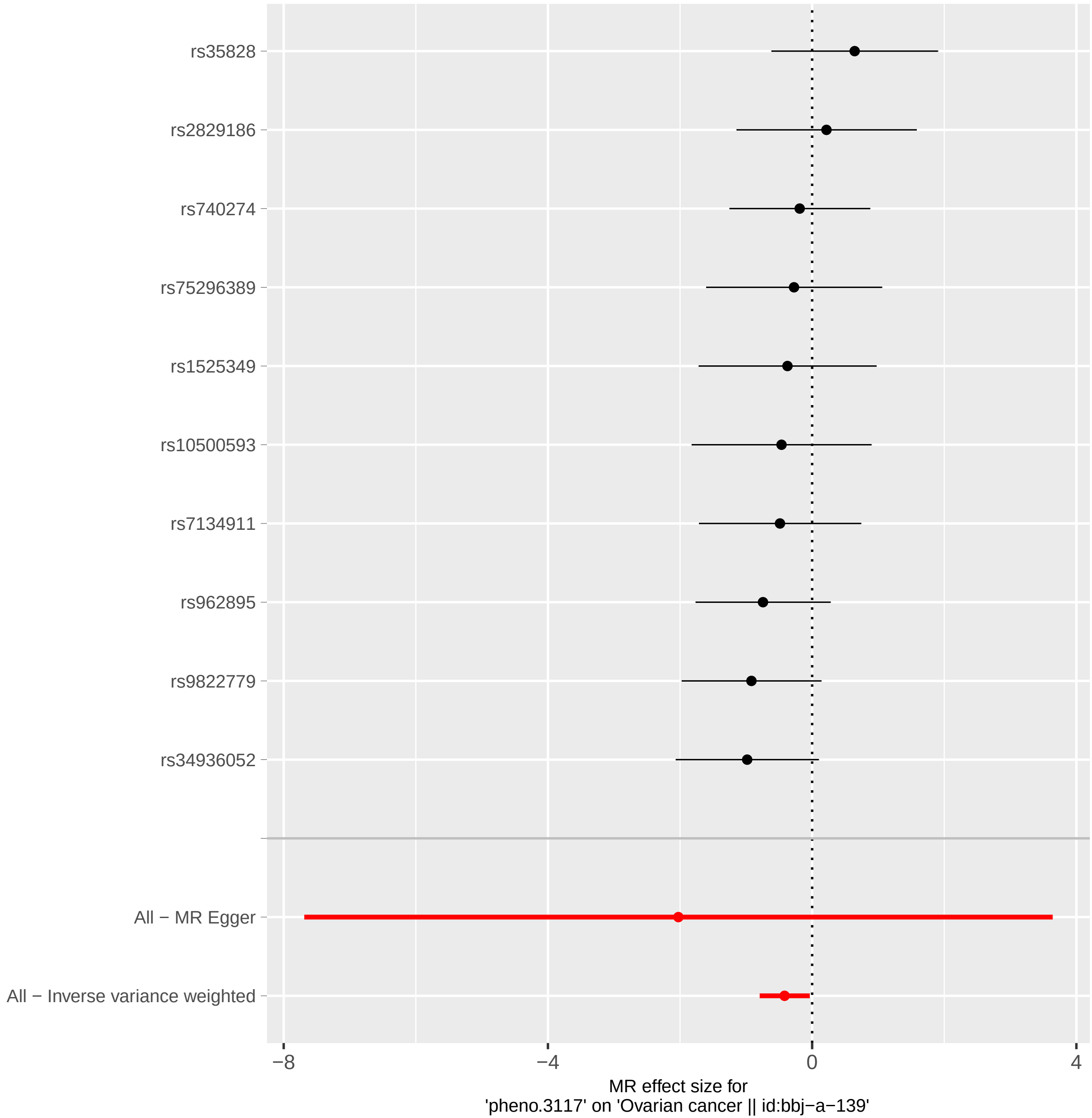

rs35828
rs2829186
rs740274
rs75296389
rs1525349
rs10500593
rs7134911
rs962895
rs9822779
rs34936052
All − MR Egger
All − Inverse variance weighted
−8
−4
0
4
MR effect size for
'pheno.3117' on 'Ovarian cancer || id:bbj−a−139'
